# Taking *Behavioral Sciences* Forward

**DOI:** 10.3390/bs7030058

**Published:** 2017-08-22

**Authors:** Scott J. Hunter

**Affiliations:** Departments of Psychiatry, Behavioral Neuroscience and Pediatrics, University of Chicago, 5841 S. Maryland Ave., MC 3077, Chicago, IL 60637, USA; shunter@uchicago.edu

It has been almost one year now since I agreed to become the Editor-in-Chief for this important open access journal, for which I have served as a member of the editorial board since its early inception. As a guest editor for two series with the journal, and a regular editor for multiple submissions, I have come to have great respect for the efforts MDPI and the *Behavioral Sciences* administrative team, the inaugural Editor-in-Chief, and the fine groups of editors and reviewers have all made to provide access to high-quality scientific papers from around the globe. I felt that coming in as the second senior editor would mean filling big shoes, as is said; and it indeed has been a significant job getting up to speed. Nonetheless, I am proud of the work that we have done to continue to take *Behavioral Sciences* forward, and to help make it a more significant option for open access publication of research addressing the cognitive and behavioral sciences.

As I think about goals for the near future, I find myself increasingly considering ways to enhance the focus and quality of the submissions received. A journal such as this, with a broad based interest in the intersection of cognitive science, behavioral medicine, social science, public health, and neuroscience, is one poised to enhance and extend ways of thinking about the individual and community simultaneously; to consider how to understand what fosters learning, memory, and problem solving, so that these capacities may be strengthened and more effectively utilized to address the needs and challenges of a diverse world. Utilizing new knowledge regarding capability and capacity across animal species, as a tool for better understanding human potential, is a secondary emphasis; one that extends our ability to think well about the interrelationships we hold with our environment, and our evolutionary history. Special Issues that seek to address complexities that arise in response to intersectional identities, and the impact that global interactions have on these challenges and opportunities, are particularly of interest. Thinking both interdisciplinarily and transdisciplinarily are equally needed as we continue to build our understanding of what it means to be evolving and growing in the 21st century.

Over the long term, I believe the main task for *Behavioral Sciences* is to become highly regarded as a home for scientific work of rigor and excellence. This journal most attracted me because it represented in its early development a setting for exploration of the multiple paths that lead to an understanding of behavior. It remains that, but can be so much more. Our task, those of us on the editorial team and those providing the scientific reports for publication, is mutual—to define more directly a focus on how behavior develops and is guided, across levels of organization and system. And to consider directly the socioeconomic, cultural, environmental, and epigenetic factors that transact and influence how we think, feel, and choose the paths that define us.

I look forward to your contributions to this adventure.

